# Anthracyclines-induced cardiotoxicity in patients with early breast cancer carrying germline BRCA1/2 mutation: the BRCAN study

**DOI:** 10.1093/oncolo/oyae299

**Published:** 2024-11-19

**Authors:** Alfonso Cortés-Salgado, Juan José Serrano, David Cordero Pereda, Miriam Menacho, José Manuel Del Rey, Laura del Campo-Albendea, Cristina Saavedra, Jesús Chamorro, Diana Rosero, Pilar Sotoca, Carmen Guillén-Ponce, Eva Guerra, María Fernández-Abad, Elena López-Miranda, Noelia Martínez-Jáñez, María Gion, María Teresa Salazar, Pilar Agudo-Quílez, Pilar Garrido, Gonzalo Luis Alonso Salinas

**Affiliations:** Medical Oncology Department, Hospital Universitario Ramón y Cajal (IRYCIS), 28034 Madrid, Spain; Medical Oncology Department, Grupo Vithas Madrid, 28043 Madrid, Spain; Cardiology Department, Hospital Universitario Ramón y Cajal (IRYCIS), 28034 Madrid, Spain; Clinical Biochemistry Department, Hospital Universitario Ramón y Cajal (IRYCIS), 28034 Madrid, Spain; Clinical Biochemistry Department, Hospital Universitario Ramón y Cajal (IRYCIS), 28034 Madrid, Spain; Biostatistics Unit, Hospital Universitario Ramón y Cajal (IRYCIS). CIBERESP, ISCIII, 28029 Madrid, Spain; Medical Oncology Department, Hospital Universitario Ramón y Cajal (IRYCIS), 28034 Madrid, Spain; Medical Oncology Department, Hospital Universitario Ramón y Cajal (IRYCIS), 28034 Madrid, Spain; Medical Oncology Department, Hospital Universitario Ramón y Cajal (IRYCIS), 28034 Madrid, Spain; Medical Oncology Department, Hospital Universitario Ramón y Cajal (IRYCIS), 28034 Madrid, Spain; Medical Oncology Department, Hospital Universitario Ramón y Cajal (IRYCIS), 28034 Madrid, Spain; Medical Oncology Department, Hospital Universitario Ramón y Cajal (IRYCIS), 28034 Madrid, Spain; Medical Oncology Department, Hospital Universitario Ramón y Cajal (IRYCIS), 28034 Madrid, Spain; Medical Oncology Department, Hospital Universitario Ramón y Cajal (IRYCIS), 28034 Madrid, Spain; Medical Oncology Department, Hospital Universitario Ramón y Cajal (IRYCIS), 28034 Madrid, Spain; Medical Oncology Department, Hospital Universitario Ramón y Cajal (IRYCIS), 28034 Madrid, Spain; Medical Oncology Department, Hospital Universitario Ramón y Cajal (IRYCIS), 28034 Madrid, Spain; Cardiology Department, Hospital Universitario Ramón y Cajal (IRYCIS), 28034 Madrid, Spain; Medical Oncology Department, Hospital Universitario Ramón y Cajal (IRYCIS), 28034 Madrid, Spain; Cardiology Department, Hospital Universitario de Navarra, 31008 Pamplona (Navarra), Spain; Navarrabiomed, IdiSNA, 31008 Pamplona (Navarra), Spain; Department of Health Sciences, Universidad Pública de Navarra (UPNA), 31008 Pamplona (Navarra), Spain

**Keywords:** breast cancer, anthracyclines, cardiotoxicity, BRCA1/2, cardio-oncology

## Abstract

**Background:**

BRCA1/2 genes play a critical role in genome stability and DNA repair. In animal models, loss of cardiomyocyte-specific BRCA1/2 is associated with DNA damage, apoptosis, cardiac dysfunction, and mortality following anthracycline exposure. However, whether these preclinical findings translate to humans remains unclear.

**Objective:**

Assess the impact of germline BRCA1/2 (gBRCA1/2) status on anthracyclines-induced cardiotoxicity (AIC) in patients with early breast cancer and no prior anti-HER2 therapy.

**Methods:**

This single-center retrospective/prospective cohort study focused on early breast cancer patients, treated with anthracycline-based chemotherapy in the neo/adjuvant setting, no prior anti-HER2 therapy, and known gBRCA1/2 status, normal baseline left ventricular ejection fraction (LVEF), and no previous cardiovascular disease. Follow-up assessments involved myocardial dysfunction blood biomarkers (MDBB), transthoracic echocardiography (TTE), and quality of life (QoL) questionnaires. The primary objective was LVEF changes comparing BRCA1/2 mutation carriers (gBRCA1/2m) vs non-carriers (gBRCA1/2wt). Secondary objectives included differences in MDBB and QoL.

**Results:**

A total of 137 patients were included (103 gBRCA1/2wt and 34 gBRCA1/2m). Baseline characteristics were similar between groups. Compared to baseline, LVEF% reduction was −4.7[−12.0, 0.0] vs −9.5[−18.0, −5.0] in gBRCA1/2wt vs gBRCA1/2m, (*P* = .027). After adjusting for confounders, the difference in reduction in LVEF remained statistically significant at −4.5 [95%CI, −8.6, −0.4; *P* = .032]. No differences between MDBB (C-reactive protein, hsTnI, NT-proBNP, D-Dimer, ST-2, or Galectine-3) or QoL (MLHFQ and EQ5-D index) were detected.

**Conclusions:**

gBRCA1/2m patients could represent a higher-risk population for AIC. gBRCA1/2 status should be one of the factors to consider in deciding on adjuvant anthracycline necessity. This population could benefit from a cardio-oncology closer follow-up and cardioprotective strategies.

## Graphical Abstract

**Graphical Abstract:**
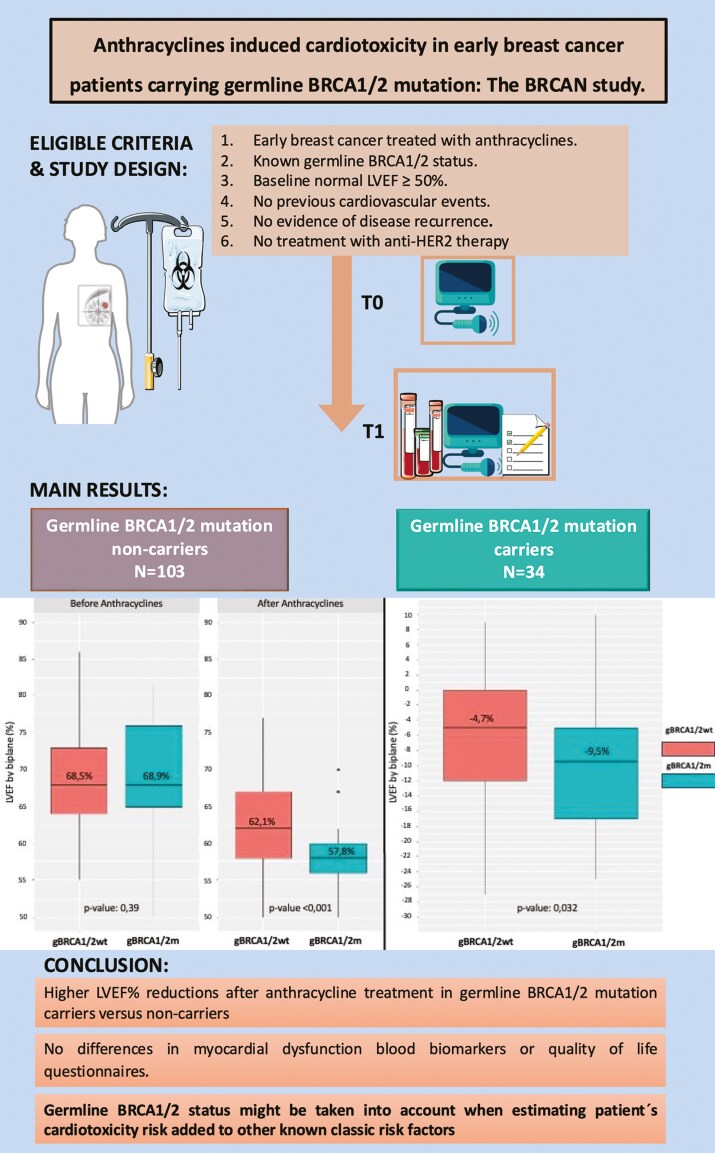

**Implications for practice**

This study underscores critical considerations for HER2-negative early breast cancer treatment: (1) genetic screening: conduct pre-anthracycline therapy germline BRCA1/2 screening to identify high-risk patients for potential cardiotoxicity; (2) informed decisions: evaluate anthracycline necessity based on genetic profiles, particularly for germline BRCA1/2 mutation carriers (gBRCA1/2m); (3) surveillance: closely monitor gBRCA1/2m patients using myocardial biomarkers and echocardiography for early detection of cardiotoxic effects; and (4) cardioprotective measures: employ targeted cardioprotective strategies during anthracycline therapy for gBRCA1/2m patients. In summary, recognizing germline BRCA1/2 status enables a personalized approach, prioritizing genetic information and proactive cardio-oncology for safer HER2-negative early breast cancer treatment.

## Introduction

According to the latest report from the European Commission and the International Agency for Research on Cancer,^1^ breast cancer remains the most diagnosed cancer in the European Union in 2023, with an estimated 380 000 new cases and the third cause of death following lung and colorectal cancer.

Due to early detection programs, most breast cancer cases are diagnosed in the early setting where anthracyclines continue to play a pivotal role.^2^ This importance is based on the significant improvements in recurrence-free survival and overall survival demonstrated by anthracyclines in this scenario.^3^

However, anthracyclines produce a very well-known, dose-dependent, and potentially irreversible anthracyclines-induced cardiotoxicity (AIC).^4^ AIC presents with a variable clinical spectrum, ranging from an asymptomatic decrease in ejection fraction to congestive heart failure, and can occur early or after years from the exposure. As an example, doxorubicin is associated with a 5% incidence of CHF when a cumulative dose of 400 mg/m^2^ is reached, and higher doses (700 mg/m^2^) carry an exponential increase in risk up to 48%.^5^

The inhibition of the enzyme topoisomerase-2beta (Top2β) has been proposed as the main cause of AIC. In humans, there are 2 types of topoisomerase enzymes, topoisomerase-2alfa (Top2α) and Top2β. Both have the function of unwinding the DNA double-strand during cell replication and transcription. Top2α is predominantly found in proliferating cells, and its inhibition produces the anticancer effect of anthracyclines. In contrast, Top2β is mainly found in quiescent cells, including cardiomyocytes. The binding of anthracycline to Top2β changes its conformation and impedes its function, leading to an accumulation of double-stranded DNA errors and consequently, the death of the cardiomyocyte.^6,7^

BRCA1 and BRCA2 genes are tumor suppressor genes that encode proteins crucial for the homologous recombination system or double-strand DNA damage repair process.^8^ Around 15% of patients diagnosed with breast cancer carry a germline heterozygous mutation in the BRCA1 or BRCA2 genes.

The importance of the BRCA1 and BRCA2 genes in DNA repair and maintenance of cardiac function after exposure to an ischemic or genotoxic insult has been widely studied in murine models. One of these works shows that, after treatment with doxorubicin, BRCA2 homozygous knock-out^(−/−)^ mice presented a greater number of double-stranded DNA errors, a lower capacity for their repair and a greater p53-mediated death of the cardiomyocyte in comparison with control mice.^9^(p2)

Similarly, mice with BRCA1 knock-out^(−/−)^ presented less repair of double-stranded DNA defects, an accumulation of DNA damage as well as greater apoptosis and cardiac dysfunction compared with wild-type (WT) BRCA1 mice.^10^(p1) Interestingly, in this study, 20% of WT mice died, in contrast to around 95% of BRCA1^(−/−)^ and 60% of the BRCA1 heterozygous knock-out^(+/−)^ suggesting some degree of cardiotoxicity also in heterozygous animals.

To date, if these preclinical findings translate to humans is still a matter of controversy, since several studies have shown conflicting results(^,11,12^(p2)^13^).

Here we show the results of BRCAN, a single center retrospective/prospective cohort study including patients diagnosed with HER2-negative early breast cancer treated with anthracycline-based chemotherapy and known BRCA1/2 status with the aim to comprehensively address the cardiac function assessed by blood biomarkers, imaging and QoL questionnaires.

## Methods

### Study design and procedures

This is a retrospective/prospective cohort study including patients diagnosed with early breast cancer treated with neo/adjuvant anthracycline-based chemotherapy, separated into two cohorts: gBRCA1/2m and gBRCA1/2wt.

The main inclusion criteria were: (1) known gBRCA1/2 status (gBRCA1/2m vs gBRCA1/2wt, (2) normal LVEF ≥ 50% before anthracyclines exposure, (3) previous significant cardiovascular events not related to anthracyclines exposure were excluded, (4) no prior anti-HER2 therapy, and (5) no evidence of disease recurrence.

For the purpose of this study, we first identified in our database patients diagnosed with hereditary breast and ovarian cancer syndrome (gBRCA1/2m) that were tested following Spanish Society for Medical Oncology (SEOM) criteria.^14,15^ Germline testing was carried out using next-generation sequencing (NGS) analysis on peripheral blood samples. Pathogenic and likely pathogenic variants were validated using Sanger sequencing. Analyzed variants were classified according to American College of Medical Genetics and Genomics criteria and classified into five classes: benign (class I), likely benign (class II), variant of uncertain significance (VUS, class III), likely pathogenic (class IV), and pathogenic (class V).^16^

Then, we matched (1:3) with non-carriers considering the time from the anthracycline exposure. After inclusion, all patients were evaluated once with myocardial dysfunction blood biomarkers (MDBB), TTE, and QoL questionnaires simultaneously.

The retrospective part of the study refers to the moment in which each patient was diagnosed with cancer, the standard treatment and (which included anthracycline-based chemotherapy) baseline TTE were performed. Therefore, epidemiological data, clinical, and pathological tumor characteristics, and information regarding local and systemic treatments were retrospectively collected. The prospective part of the study refers to the current recruitment, where the second TTE, MDBB analysis, and QoL questionnaires were performed.

For MDBB analysis, 3 blood samples from each patient were obtained at the time of the inclusion: 2 gel and clot activator tubes for serum collection and one sodium citrate tube for plasma analysis. Blood samples underwent centrifugation at 3500 revolutions per minute (rpm) at 7°C for 10 minutes. A 500 µL aliquot of the serum fraction was stored at −80°C until all samples were collected. Those aliquots were thawed at room temperature and homogenized in the vortex mixer for 10 seconds before being employed for GDF-15, ST-2 and Galectina-3 determination. High sensitivity troponin I (hsTnI), C-reactive protein (CRP), and D-dimer were analyzed immediately after sample obtaining and processing.

The determination of hsTnI, NTproBNP, and galectin-3 was performed by quantitative Chemiluminescence Microparticle Immunoassay (CMIA) using the Alinity i analyzer (Abbott). hsTnI and galectin-3 were expressed as nanograms per milliliter (ng/mL) and NTproBNP was expressed as picograms per milliliter (pg/mL). For PCR analysis, turbidimetric immunoassay in the Alinity c analyzer (Abbott) was employed, and results were in milligrams per deciliter (mg/dL). D-dimer was also analyzed by turbidimetric immunoassay in the CS-5100 system (Sysmex—Siemens) and results were expressed as ng/mL. SLT-2 was quantified by enzyme-linked immunosorbent assay (ELISA) with Dynex (Thermo Fischer Scientific) and expressed as (pg/mL). GDF-15 was analyzed by electrochemiluminescent assays (ECLIA) with Cobas e601 (Roche), and results were expressed as pg/mL.

We performed a comprehensive transthoracic echocardiography (TTE) approach employing the Philips Epiq X5 transducer (Philips Healthcare). All measurements were performed during sinus rhythm according to the European Association of Echocardiography recommendations.^17^ Five consecutive cardiac cycles were acquired from each view and digitally stored. Two-dimensional apical 2-, 3-, and 4-chamber views for 5 cycles were acquired and stored for later analysis. LVEF was analyzed by Simpson’s method. The sonographer was blinded for the study groups.

QoL was assessed through the Minnesota Living with Heart Failure Questionnaire (MLHFQ) and the EQ5D questionnaire. MLHFQ is a patient-reported outcomes questionnaire specifically designed to assess QoL in patients with heart failure. While que EQ5D questionnaire is a self-completion generic questionnaire to measure QoL, applicable to a wide range of health conditions.

The study was performed in accordance with Good Clinical Practice guidelines and the World Medical Association Declaration of Helsinki,^18^ and it was approved by the institutional review board of Ramón y Cajal University Hospital (Madrid) (ACTA 397; Código 174-020). All patients provided written informed consent before any procedure.

### Statistical analysis

Data were analyzed with STATA v17.0. Descriptive statistics for the main population characteristics were reported as either median (first quartile; third quartile) or mean (± standard deviation) for continuous variables and absolute and relative frequency for categorical variables. The relationship between variables was assessed using the *X*^*2*^-squared, *t*-Student, or Mann-Whitney test, as appropriate. Lineal regression was used to analyze the primary and secondary endpoints. Sample was calculated to detect a difference between groups of 5% in terms of LVEF, with a power of 0.89. A *P* value of<.05 was considered statistically significant.

### Study endpoints

The primary endpoint of the study was to assess the %LVEF reduction from baseline in gBRCA1/2m vs gBRCA1/2wt after treatment with anthracyclines.

Secondary endpoints were to evaluate the difference in MDBB and QoL in gBRCA1/2m vs gBRCA1/2wt.

## Results

### Populations characteristics and treatments received.

A total of 137 patients were included in the study, 103 gBRCA1/2wt and 34 gBRCA1/2m enrolled from December 2020 to May 2022. There were 19 BRCA1 patients (55.9%) and 15 BRCA2 patients (44.1%). Mean age was 49.1 and 43.2 years respectively (with a standard deviation (SD) of 29.3 and 10.2 years), with no differences between them (*P* = .13).

There were no differences between both groups in tumor characteristics. Right breast was affected in 49.5% of gBRCA1/2wt and 50% of gBRCA1/2m patients (*P* = .94). Using the AJCC 8th edition TNM, there were no differences in T-stage (*P* = .73) or in N-stage (*P* = .30). Stage IIA was the most represented stage, in 38.4% of gBRCA1/2wt and in 44.1% of gBRCA1/2m cases, respectively (*P* = 0.33). According to the biology of the disease, there were more triple-negative tumors in gBRCA1/2m (58.8%) and more luminal tumors within gBRCA1/2wt patients (58.2%), without reaching significant differences between groups (*P* = .08).

Since gBRCA1/2m patients had a higher frequency of risk-reducing mastectomies, more patients in the gBRCA1/2wt group received breast radiotherapy at standard doses (85.45% vs 64.7%, *P* = .004). However, there were no differences between patients who received radiotherapy on the left side (40.8% vs 29.4%, *P* = .12). Importantly, 27(79.4%) of gBRCA1/2m patients received prophylactic bilateral salpingoophorectomy and 12 (44.4%) of them were premenopausal at the time of surgery.

The most widely used anthracycline was adriamycin: 93.2% in gBRCA1/2wt and 91.2% in gBRCA1/2m (*P* = .69). Importantly, there were no differences in the mean doses of Adriamycin: 237.4 mg/m^2^ vs 250.8 mg/m^2^ (SD 41.5 vs 35.8, *P* = .11); nor Epirubicin: 325 mg/m^2^ vs 400 mg/m^2^ (SD 77.9 vs 124.9, *P* = .15) between groups.

There were no differences in median time from the exposure to anthracyclines to inclusion in the study, being 7.5 years for gBRCA1/2wt and 8.3 years for gBRCA1/2m (SD 4.6 and 5.2, respectively, *P* = .20). All baseline characteristics are summarized in Table 1.

**Table 1.** Baseline characteristics

Characteristic	gBRCA1/2wt (103)	gBRCA1/2m (34)	*P*-value
*Demographics*			
Age, mean (SD)	49.1 (29.3)	43.2(10.2)	.13
Race			
Caucasian	94 (91.3)	34 (100.0)	.20
Hispanic	8 (7.7)	0 (0.0)	
BMI, mean (SD)	25.7 (4.4)	24.5 (4.5)	.26
*Tumor characteristics*			
Laterality			
Bilateral	2 (1.9)	1 (2.9)	.94
Right	51 (49.5)	17 (50.0)	
Left	49 (47.6)	16 (47.1)	
*Tumor stage* ^*^			
I	12 (11.8)	8 (23.5)	.33
IIA	40 (38.4)	15 (44.1)	
IIB	30 (29.4)	6 (17.6)	
IIIA	13 (12.7)	4 (11.8)	
IIIC	7 (6.9)	1 (2.9)	
*Tumor size* ^*^			
T1	34 (33.0)	14 (41.2)	.74
T2	57 (55.3)	18 (52.9)	
T3	10 (9.7)	2 (5.9)	
T4	1 (1.0)	0 (0.0)	
*N stage* ^*^			
N0	37 (35.9)	18 (52.9)	.30
N1	45 (43.7)	11 (32.3)	
N2	9 (8.7)	4 (11.8)	
N3	8 (7.8)	1 (2.9)	
Nx	4 (3.9)	0 (0.0)	
Luminal disease	60 (58.2)	14 (41.2)	.083
Triplenegative	43 (41.7)	20 (58.8)	
HER2-positive	3 (2.9)	0 (0.0)	
*Therapy characteristics*			
Radiotherapy	88 (85.4)	22 (64.7)	.004
Radiotherapy on left side	42 (40.8)	10 (29.4)	.12
Adriamycin	97 (94.2)	31 (91.2)	.69
Total dose adriamycin (mg/m^2^), mean (SD)	237.4 (41.5)	250.8 (35.8)	.11
Epirubicin	6 (5.8)	3 (8.8)	.54
Total dose epirubicin (mg/m^2^), mean (SD)	325 (77.9)	400 (124.9)	.15
Exposition time (years), mean (SD)^^^	7.5 (4.6)	8.3 (5.2)	.20
*Other drugs use*			
Cyclophosphamide	103 (100.0)	32 (94.1)	.060
Paclitaxel	86 (83.5)	24 (70.6)	.10
Docetaxel	13 (12.6)	1 (2.9)	.11
Carboplatin	14 (13.6)	4 (11.8)	.78
Other	19 (18.4)	9 (26.4)	.31

Numbers refer to *n* with percentage in parenthesis, unless otherwise specified.

^*^According to the AJCC 8th edition TNM.

^^^Time elapsed from when the patients received the anthracyclines until they were included in the study.

‘Include capecitabine, 5-florouracyl, bevacizumab, pembrolizumab, atezolizumab, ipatasertib, palbociclib.

Abbreviations: BMI, body mass index, gBRCA1/2wt, patients without mutation in BRCA1/2 germline, gBRCA1/2m, patients with mutation in BRCA1/2 germline; SD, standard deviation.

**Figure 1.**
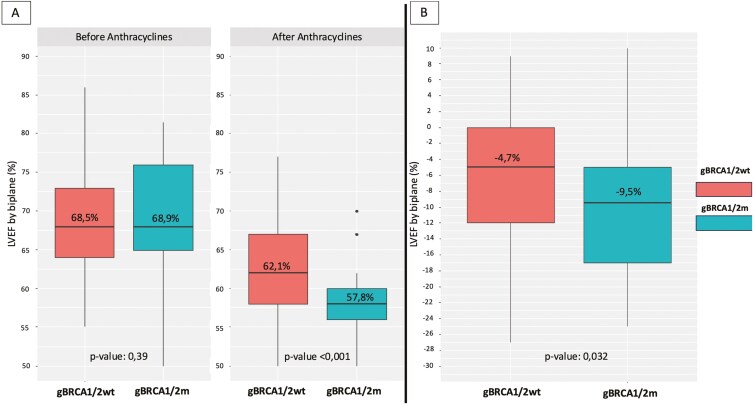
(A) Left ventricular ejection fraction before and after anthracyclines. (B) Average reduction in left ventricular ejection fraction after anthracyclines.

The mean baseline percentage of LVEF was 68.5% in gBRCA1/2wt and 68.9% in gBRCA1/2m (*P*=.38) (Figure 1A). Regarding comorbidities, hypertension was more commonly reported in gBRCA1/2wt (13.6% vs 0%, *P* = .023) as well as hypercholesterolemia (16.5% vs 2.9%, *P* = .041). Importantly, there were no differences in the other variables such as diabetes mellitus, use of antihypertensive, hypolipidemic, or antiaggregant agents. There were also no differences in life habits such as daily physical exercise (*P* =.28), alcohol consumption (*P* =.91), or smoking habit (*P* = .82). The personal medical history and patients’ habits are summarized in Table 2.

**Table 2.** Medical history and lifestyle.

Characteristic	gBRCA1/2wt	gBRCA1/2m	*P*-value
*Personal medical history*			
Initial LVEF % (SD)	68.5 (6.7)	68.9 (8.3)	.38
Hypertension	14 (13.6)	0 (0.0)	.023
Diabetes mellitus	5 (4.8)	0 (0.0)	.19
Hypercholesterolemia	17 (16.5)	1 (2.9)	.041
Antihypertensive agents use	14 (13.6)	1 (2.9)	.11
Hypolipidemic agents use	25 (24.3)	6 (17.6)	.60
Antiplatelet agent use	2 (1.9)	2 (5.8)	.20
*Daily physical exercise (measured as aerobic physical exercise per week)*			
High (>300 minutes)	7 (6.8)	1 (2.9)	.29
Medium (150-300 minutes)	33 (32.0)	17 (50.0)	
Low (<150 minutes)	39 (37.9)	10 (29.4)	
Inactive (no physical activity)	24 (23.3)	6 (17.6)	
*Alcohol consumption*			
Daily	2 (1.9)	1 (2.9)	.91
Weekly	10 (9.7)	3 (8.9)	
Occasional	45 (43.7)	13 (38.2)	
None	45 (43.7)	17 (50.0)	
*Smoke habit*			
Smoker	17 (17.2)	5 (14.7)	.82
Ex-smoker	35 (35.4)	14 (41.2)	
Non-smoker	47 (47.5)	15 (44.1)	

Numbers refer to *n* with percentage in parenthesis, unless otherwise specified.

Abbreviations: gBRCA1/2m, patients with mutation in BRCA1/2 germline; gBRCA1/2wt, patients without mutation in BRCA1/2 germline; LVEF: left ventricular ejection fraction; SD, standard deviation.

### Left ventricular ejection fraction after exposure

After receiving anthracyclines, a difference between the means of LVEF percentage of gBRCA1/2wt and gBRCA1/2m was detected: 62.1% vs 57.8% [95%CI 1.85, 6.7, *P* < .001] (Figure 1A). Compared to baseline, %LVEF reduction was −4.7 [−12.0, 0.0] in gBRCA1/2wt group and −9.5 [−18.0, −5.0] in gBRCA1/2m group [−4.3, 95%CI, −8.1, −0.5, *P* = .027], showing a greater average %LVEF reduction in BRCA1/2m group (*P* = .032) after anthracyclines (Figure 1B). To correct for possible confounding factors, we performed a regression model using smoking status and the use of antihypertensive and hypolipidemic agents’ variables. After correction, this difference in average %LVEF reduction in the gBRCA1/2m group compared to the gBRCA1/2wt was −4.5, maintaining statistical significance [95%CI, −8.6, −0.4, *P* = 0.03]. We also identified that 38.8% of gBRCA1/2wt vs 58.8% of patients in gBRCA1/2m group experienced a LVEF decline of ≥10% (*P* = 0.12). Interestingly, only 1.9% of gBRCA1/2wt and 8.8% of gBRCA1/2m patients experienced an LVEF reduction below 50% (*P* = .06).

This decrease in LVEF was asymptomatic in all patients.

### Myocardial dysfunction blood biomarkers

**Figure 2.**
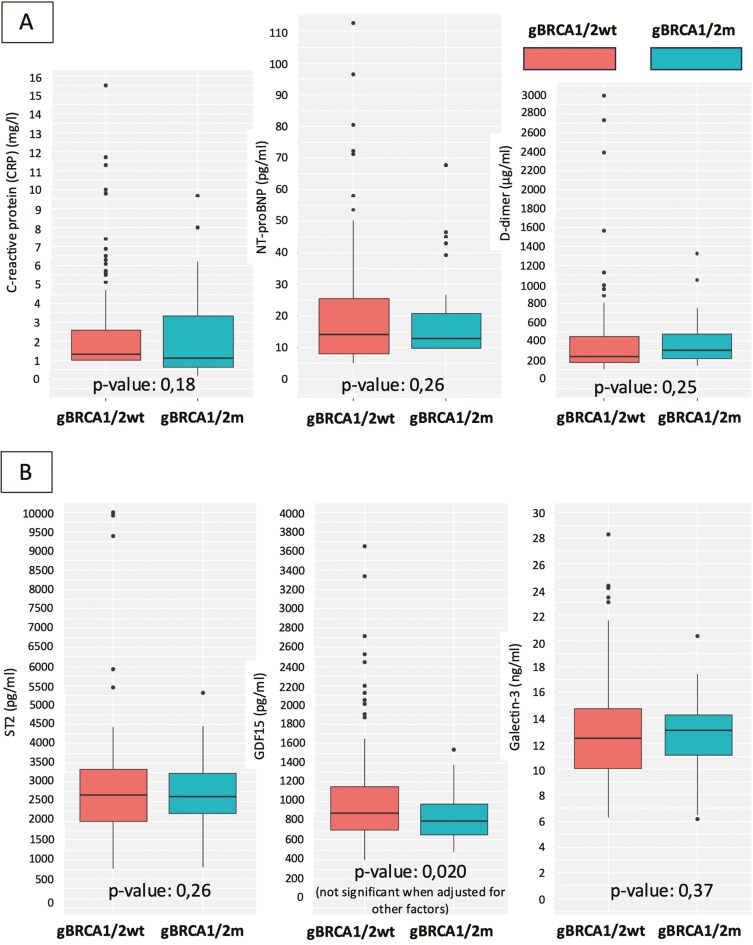
**(A)** Traditional myocardial dysfunction blood biomarkers. (B) Novel myocardial dysfunction blood biomarkers.

Interestingly, no differences were observed in MDBB between both groups. Using the gBRCA1/2wt group as a reference, the difference for CRP was −0.7 [95%CI, −2.1, 0.8, *P* = .18], for hsTnI was −0.1 [95%CI, −0.2, 0.1, *P* = .13], for N-terminal pro-B-type natriuretic peptide (NT-proBNP) was −2.4 [95%CI, −9.6, 4.8, *P* = .26] and for D-dimer was −83.2 [95%CI, −331.4, 165.0, *P* = .25]. The data are summarized in Table 3 and Figure 2A. Regarding the advanced MDBB, the difference for ST-2 was −199.2 [95%CI, −795.5, 397.0, *P* = .25], for galectin-3 was −0.3 [95%CI, −1.8, 1.2, *P* = .36]. Surprisingly, we initially observed a statistical difference in GDF-15 of −214.0 [95%CI, −418.5, 9.5, *P* = .020].

**Table 3.** MDBB values.

MDBB	gBRAC1/2wt	gBRCA1/2m	Coefficient (95% CI)	*P*-value	*P*-value (r.m.)^*^
*Traditional*					
CRP	2.9 (0.4)	2.3 (0.4)	−0.7 (−2.1, 0.8)	0.18	.66
hsTnI	0.1 (0.03)	0.0 (0.0)	−0.1 (−0.2, 0.1)	0.13	.31
NT-proBNP	21.0 (1.9)	18.6 (2.4)	−2.4 (−9.6, 4.8)	0.26	.72
D-dimer	465 (0.5)	381.8 (44.4)	−83.2 (−331.4, 165.0)	0.25	.68
*Novel*					
ST-2	2926.7 (165.0)	2727.5 (156.9)	−199.2 (−795.5, 397.0)	0.26	.75
GDF-15	1045.5 (57.4)	831.4 (45.3)	−214.0 (−418.5, 9.5)	0.020	.12
Galectin-3	12.9 (0.4)	12.7 (0.5)	−0.3 (−1.8, 1.2)	0.37	.88

Numbers refer to mean with standard deviation in parenthesis.

^*^In a regression model with the variables smoking habit and use of antihypertensive and hypolipidemic agents.

Abbreviations: CI, confidence interval; CRP, C-reactive protein; hsTnI, highly sensitive Troponin I; MDBB, myocardial dysfunction blood biomarkers; NT-proBNP, N-terminal pro B-type natriuretic peptide; r.m., regression model.

After applying a regression model adjusted for smoking status and the use of antihypertensive and hypolipidemic agents, no discernible differences were observed.

We also studied if any of MDBB correlated with LVEF decline, regardless of gBRCA1/2 status. In that respect, D-Dimer was increased in patients with FEVI decline≥10% comparing those with LVEF decline ≤10% or no decline (*P* = .053).

The data are summarized in Table 3 and Figure 2B.

### Quality of life questionnaires

**Figure 3.**
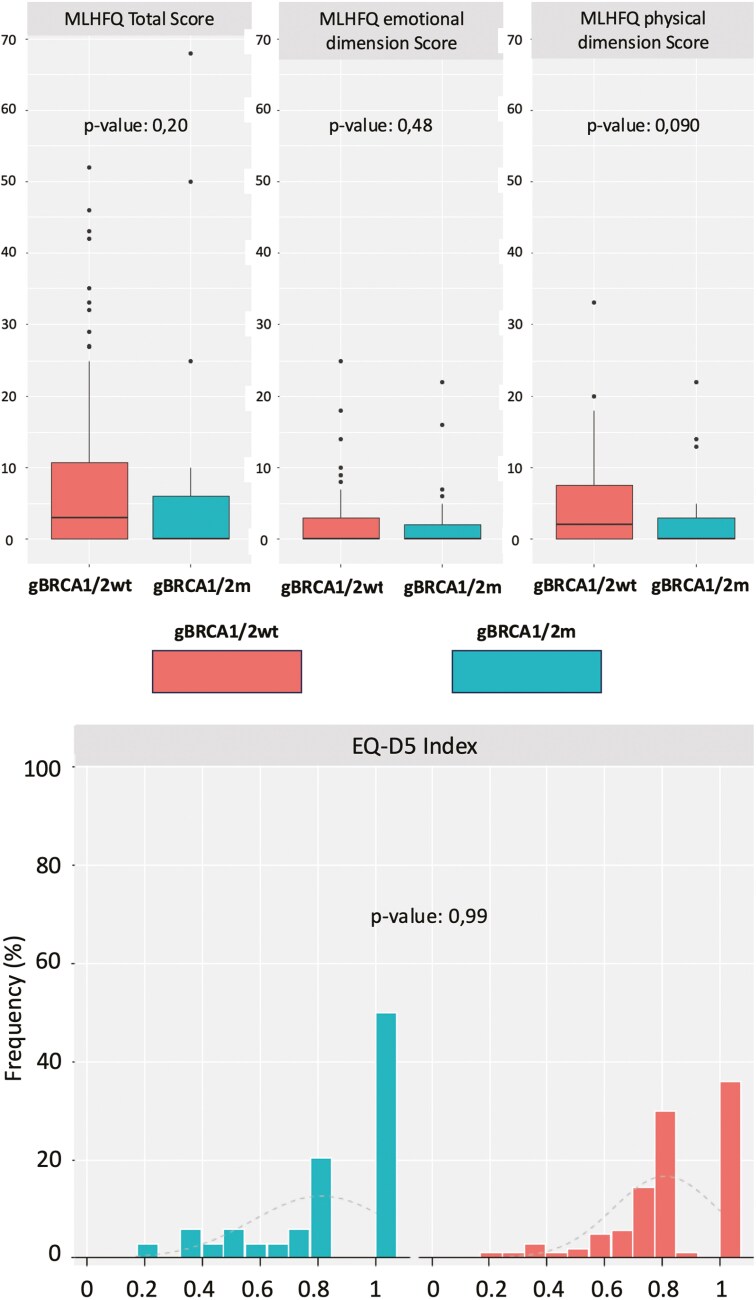
Heart-related quality of life questionnaires.

We also studied if the differences in LVEF resulted in changes in QoL. Using MLHFQ questionnaire, the difference between gBRCA1/2wt and gBRCA1/2m in the physical section was −1.7 points [95%CI, −4.2, 0.8, *P* = .09], in the emotional section was 0.03 points [95%CI, −1.6, 1.7, *P* = .48] and the overall score was −2.6 points [95%CI, −8.7, 3.5, *P* = .20]. In the EQD-5 questionnaire, the score obtained was similar [95%CI, −0.1, 0.1, *P* = .99]. We also performed a regression model that was adjusted for smoking status and use of antihypertensive and hypolipidemic agents but again, no differences were observed. There were no differences in any of QoL questionnaires comparing those patients who experienced an FEVI decline ≥10% to those with FEVI decline ≤10%, regardless of gBRCA1/2 status. The data are summarized in Table 4 and Figure 3.

**Table 4.** Results of QoL questionnaires.

MDBB	gBRAC1/2wt	gBRCA1/2m	Coefficient (95% CI)	*P*-value	*P*-value (r.m.)^*^
MLHFQ: physical	4.7 (0.6)	3.0 (0.1)	−1.4 (−4.0, 1.2)	.090	.29
MLHFQ: emotional	2.2 (0.4)	2.3 (0.8)	0.2 (−1.5, 2.0)	.48	.78
MLHFQ: overall	9.0 (1.5)	6.3 (2.7)	−1.4 (−7.7, 5.0)	.20	.67
EQ-5D	0.8 (0.2)	0.8 (0.2)	0.0 (−0.1, 0.1)	.99	.63

Numbers refer to mean with standard deviation in parenthesis.

^*^In a regression model with the variables smoking habit and use of antihypertensive and hypolipidemic agents.

Abbreviations: CI, confidence interval; MLHFQ, Minnesota Living with Heart Failure Questionnaire; QoL, health-related quality of life; r.m., regression model.

## Discussion

We conducted this single-center retrospective/prospective cohort study exploring AIC on patients with early breast cancer with known gBRCA1/2 status, with the aim to assess the impact of gBRCA1/2 status on AIC. Our results demonstrate that gBRCA1/2m patients developed a statistically significant reduction in LVEF compared to gBRCA1/2wt, even after correcting for possible confounding factors such as smoking habit or the use of hypolipidemic or antiaggregant drugs. This reduction was asymptomatic in all patients.

Baseline characteristics were similar between groups regarding tumors, treatments received, cardiovascular comorbidities, or lifestyle. One of the key clinical aspects we analyzed was radiotherapy. In our study, more patients in the gBRCA1/2wt cohort received radiotherapy in comparison with gBRCA1/2m. However, there were no differences between groups in terms of radiotherapy on the left side. We consider it important to highlight this distinction since radiotherapy on the left side is more cardiotoxic.

We also registered the rate of prophylactic bilateral salpingoophorectomy in the gBRCA1/2m cohort, which was performed in about 80% of patients. Of them, 44.4% were premenopausal at the time of surgery. It is well known that early menopause is linked to an increased risk of cardiovascular disease, mainly coronary heart disease, due to the reduction in circulating estrogen.^19^

The results of our work are in line with a study recently published.^20^ In this work, 483 patients with early breast cancer treated with anthracyclines were included and separated into 3 different cohorts depending on the results of the genetic testing (gBRCA1/2m cohort, homologous recombination repair (HRR) pathway cohort or WT cohort). The aim of the study was to investigate not only BRCA1/2 mutations but also other mutations in HRR genes and their impact on AIC. Compared to baseline, they found LVEF significant reductions by TTE after treatment with anthracyclines in gBRCA1/2m and HRR cohorts, without differences in the WT cohort. Of note, the median time between the first and second TTE was only 3.4 months, which, from our point of view, may be adequate to observe short-term changes, but insufficient to observe long-term changes.

Another prior retrospective exploratory study of 401 gBRCA1/2m patients with breast cancer showed an increased risk of heart failure based on a self-reported symptoms questionnaire compared to historical controls taken from the general population. However, the interpretation of this study is limited due to the lack of verification of the survey using objective confirmatory data such as TTE.^21^

Conversely, other trials failed to demonstrate an increased risk of AIC in this population. In a small retrospective/prospective study, TTE was performed in 39 gBRCA1/2mut and 42 gBRCA1/2wt normotensive patients previously treated with anthracyclines. The results showed no differences between groups in any of the parameters assessed by TTE. Importantly, the absence of a baseline TTE and the lack of statistical power prevents the correct analysis of this study.^11^ In addition, another study with a similar design found no differences in terms of symptomatic heart failure.^13^

More recently, another work enrolled 67 patients with early breast cancer that were classified into 3 different groups: gBRCA1/2m treated with doxorubicin, gBRCA1/2wt treated with doxorubicin and gBRCA1/2m treated with non-doxorubicin treatment and they all were assessed by TTE and cardiopulmonary exercise test. Again, no differences in LVEF nor cardiopulmonary test were seen between gBRCA1/2mut and gBRCA1/2wt in doxorubicin-treated patients. Again, the small sample sizes of groups and the deficit of baseline ETT make it difficult to draw conclusions.^12^(p2)

From our point of view, a possible explanation for the discrepancy between studies could be the retrospective nature of them, the small sample sizes, the differences in follow up, heterogeneity of the population studied (some of the studies included HER2-positive patients, usually treated with another cardiotoxic anti-HER2 therapy) and the different assessments used.

To comprehensively assess AIC, we incorporated MDBB with the aim of knowing their role in identifying patients with early dysfunction before any asymptomatic LVEF decline.^22-25^ With this in mind, we used hsTnI, NT-proBNP, D-Dimer, ST-2, Galectine-3, and GDF-15. Unfortunately, we did not see any difference suggesting no benefit of the use of MDBB in this setting. This would mean that especially in long-term survivors, a follow-up strategy based on classic cardiac biomarkers would not be useful in identifying AIC in this population.

Another interesting aspect is the lack of impact on QoL. This could be explained by the fact that only 8.8% of gBRCA1/2m and 1.9% gBRCA1/2wt patients experienced an LVEF reduction below 50% (*P* = .06). The study population might be a low-risk population for AIC due to their younger age and low prevalence of cardiovascular risk factors which might be an explanation of the small sample of patients with final LVEF reduction to less than 50%.

All these aspects lead us to the question of the 9.5% LVEF average reduction seen in the gBRCA1/2m cohort can be considered clinically meaningful. From our point of view, it is not possible to discard any potential long-term consequences of this decline initially observed. It is well-known that AIC is most likely a phenomenon characterized by a continuous progressive decline in LVEF, with many affected patients being initially asymptomatic, with clinical manifestations appearing years later, often in the context of other triggering factors.^26^

In this sense, the early detection of AIC is relevant since it contributes to a better evolution of the patients if adequate interventions are timely implemented. These interventions can include the introduction of cardioprotective agents such as angiotensin-converting enzyme inhibitors/angiotensin receptor blockers, beta-blockers, spironolactone or SGLT-2 inhibitors but also lifestyle changes such as Mediterranean diet or supervised exercise interventions.^27,28^ Noteworthy, our study results allow us to suggest that patients with BRCA1/2 mutation might be at higher-risk of greater average %LVEF reductions after treatment with anthracyclines in a population that otherwise would be considered at low risk, and therefore BRCA1/2 status might be taken into account when estimating patient´s AIC risk added to other known classic risk factors.

### Limitations of the study

This study is not exempt from limitations. First, the small sample size of the gBRCA1/2m cohort. Second, the predominantly retrospective nature of the study prevents us from accessing complete data in some cases, such as in the case of salpingo-oophorectomies or concrete radiotherapy doses, although we believe these data do not influence significantly the conclusions of our study. ETT and MDBB were not performed at the same time point in all patients but the median time from exposure to inclusion of the study was more than 7 years, which is considered sufficient for capturing early and late-onset events.^29(p4)^ Finally, we did not study pathogenic variants in any other genes involved in HRR that could play a role in AIC.

## Conclusion

We present a study with a well-characterized and comprehensively assessed early breast cancer population showing a statistically significant reduction in LVEF in gBRCA1/2 patients with breast cancer compared to gBRCA1/2wt. gBRCA1/2 status should be one of the factors to consider in the decision-making regarding the use of adjuvant anthracyclines. When used, these AIC higher-risk patients could benefit from a cardio-oncology closer follow-up and from cardioprotective strategies.

## Data Availability

The data underlying this article will be shared on reasonable request to the corresponding author.
